# Tuberculosis patients display a high proportion of CD8^+^ T cells with a high cytotoxic potential

**DOI:** 10.1111/1348-0421.12724

**Published:** 2019-07-26

**Authors:** Leslie Chávez‐Galán, Jhacqueline Illescas‐Eugenio, Magaly Alvarez‐Sekely, Renata Baez‐Saldaña, Raúl Chávez, Ricardo Lascurain

**Affiliations:** ^1^ Integrative Immunology Laboratory National Institute of Respiratory Diseases “Ismael Cosío Villegas” (INER) Mexico City Mexico; ^2^ Department of Internal Medicine, Clinic 1 Mexican Social Security Institute (IMSS) Orizaba Veracruz Mexico; ^3^ Department of Hematology National Institute of Cancerology (INCAN) Mexico City Mexico; ^4^ Oncologic Pulmonology Clinic, National Institute of Respiratory Diseases “Ismael Cosío Villegas” (INER) Mexico City Mexico; ^5^ Department of Biochemistry, Faculty of Medicine National Autonomous University of Mexico (UNAM) Mexico City Mexico; ^6^ Homeopatic National Hospital Chimalpopoca135 06800 Mexico City Mexico

**Keywords:** cytotoxicity by flow cytometry, high cytotoxic ability, Th1/Th2 imbalance, tuberculosis

## Abstract

Tuberculosis (TB) is an infectious disease caused by *Mycobacterium tuberculosis* (*Mtb*) and remains a major cause of morbidity and mortality worldwide. In the host's immune response system, T cells play a critical role in mediating protection against *Mtb* infection, but the role of CD8^+^ T cells is still controversial. We evaluated the phenotypical characterization and cytotoxic ability of CD8^+^ T cells by flow cytometry‐based assay. Cytokine levels in serum were measured by multiplex cytokine assay. Our data show that cells from TB patients have an increased percentage of peripheral blood CD8^+^αβ^+^ T (*p* = 0.02) and CD56^+^CD8^+^ T (*p* = 0.02) and a decreased frequency of NKG2D^+^CD8^+^ T (*p* = 0.02) compared with healthy donors. Unlike CD8^+^ T cells from healthy donors, CD8^+^ T cells from TB patients exhibit greater cytotoxicity, mediated by HLA class I molecules, on autologous monocytes in the presence of mycobacterial antigens (*p* = 0.005). Finally, TB patients have a proinflammatory profile characterized by serum high level of TNF‐α (*p* = 0.02) and IL‐8 (*p = *0.0001), but, interestingly, IL‐4 (*p* = 0.002) was also increased compared with healthy donors. Our data show evidence regarding the highly cytotoxic status of CD8^+^ T cells in *Mtb* infection. These cytotoxic cells restricted to HLA‐A, B, and C could be used to optimize strategies for designing new TB vaccines or for identifying markers of disease progression.

**Abbreviations**
7‐AAD: 7‐amino actinomycin D
BCG: Mycobacterium bovis bacillus Calmette‐Guérin
BSA: Bovine serum albumine
CFAE: Culture filtrate antigen extract
CFSE: carboxyfluorescein diacetate succinimidyl ester
EDTA: Ethylenediaminetetraacetic acid
HIV: Human immunodeficiency virus
HLA: Human Leukocyte Antigen
HLA‐A,B,C: Human Leukocyte Antigen Class I
IFN‐γ: Interferon gamma
IL: Interleukine
IL‐1β: Interleukin 1 beta
IL‐4: Interleukin 4
IL‐8: Interleukin 8
IL‐10: Interleukin 10
IO: ionomycin
Iqr: interquartile range
mAb: monoclonal antibodies
Mtb: Mycobacterium tuberculosis
MHC: Mayor Histocompatibility Complex
NK: Natural killer cell
PBMC: Peripheral blood mononuclear cells
PBS: Phosphate‐buffered saline
PMA: phorbol 12‐myristate 13‐acetate
PPD: purified protein derivative test
TB: Tuberculosis
TCR: T cell Receptor
TNF: Tumour Necrosis Factor

## 1 INTRODUCTION

Tuberculosis (TB) is an infectious disease whose causative agent, *Mycobacterium tuberculosis* (*Mtb*), an intracellular bacillus, is primarily transmitted via aerosolized droplets into the lungs, where it establishes infection. TB is a leading cause of death worldwide from a single infectious agent; in 2016, the World Health Organization estimated that 10.4 million people fell ill with TB, resulting in 1.7 million deaths.^1^

The protective immune response against *Mtb* requires a balance between innate and adaptive immunity. The primary progressive and reactivated TB in human immunodeficiency virus (HIV)‐infected individuals has demonstrated convincingly that CD4^+^ T cells are important in the control of *Mtb* infection.^2^ However, mice with depleted CD8^+^ T cells have shown that survival time is also affected during *Mtb* infection.^3^

As was reviewed by Chávez‐Galán et al^4^ CD8^+^ T cells have a cytotoxic function, and their ability to induce target cell death is mainly by recognition of the human leukocyte antigen class I (HLA‐A, B, or C)‐restricted peptide antigens. Besides classic CD8^+^ T cells, there are CD8^+^ T‐cell subpopulations that coexpress markers of natural killer (NK) cells, such as NKG2D, CD16, and CD56, and a characteristic of these CD8^+^ T cells is their strong ability to secrete cytokines, such as IFN‐γ, or their more potent cytotoxicity.^5^, ^6^, ^7^

In the context of mycobacterial infection, it has been shown that antigen presentation via MHC class I by classic CD8^+^ T cells is an essential component of resistance to *Mtb* in mice.^8^ Moreover, Jacobsen et al^9^ demonstrated a clonal expansion of CD8^+^ T cells in childhood TB. In fact, mycobacterial antigen (Ag)‐specific CD8^+^ T cells are important in measuring the protective capacity of new TB vaccines in clinical trials.^8^, ^10^, ^11^, ^12^ Likewise, some immunogenic peptide antigens have been proposed as immunotherapy candidates for inducing cytotoxic activity in TB drug‐resistant patients.^13^ Thus, it has now been accepted that CD8^+^ T cells play a critical role in the immune response against *Mtb* infection, as reviewed by Lin and Flynn.^14^

Regarding the murine model of TB, several authors have shown that mycobacterial Ag‐specific T cells, which are IFN‐γ producers, are constantly moving from lymphoid tissue to the lungs during *Mtb* infection, suggesting that effector CD4^+^ and CD8^+^ T cells can circulate through the bloodstream.^15^, ^16^ Accordingly, we need to increase our understanding of the role that CD8^+^ T cells play in TB, because this cell population could be relevant for the development of new vaccines and immunotherapies, and could possibly be used as a marker of disease progression.

Here, we investigated whether adult patients with TB (TB patients) had increased cytotoxic T‐cell frequency in their peripheral blood, and what their ability to induce cytotoxicity on target cells was. In addition, we examined the expression of αβ and γδ T‐cell receptor (TCR) chains, as well as other molecules involved in the activation of a cytotoxic function, such as NKG2D and CD56, on CD8^+^ T‐cell surfaces in TB patients. Finally, we evaluated the cytotoxic ability of CD8^+^ T cells in a coculture with autologous CD14^+^ cells in the presence of a mycobacterial antigen using a flow cytometry‐based cytotoxic assay.

## 2 MATERIAL AND METHODS

### 2.1 Study population

The study population consisted of 33 patients with active pulmonary TB; diagnosis of TB was based on clinical history, physical examination, chest X‐rays, identification of acid‐fast bacilli in sputum, and positive culture to *Mtb*. Patients with comorbidities, such as HIV infection, malignant diseases, diabetes mellitus, chronic renal failure, and liver cirrhosis, were excluded. Patients with extrapulmonary TB, as well as patients diagnosed and treated previously for TB and multidrug‐resistant TB, were also excluded. Our study subpopulation was confined strictly to patients with active pulmonary TB, sensitive to treatment, and diagnosed for the first time.

The healthy control group consisted of 29 healthy volunteers who had received the *Mycobacterium bovis* bacillus Calmette–Guérin (BCG) vaccine: The Mexican vaccination scheme includes a single dose of BCG at birth. Clinical histories and physical examinations confirmed that control group volunteers had no respiratory symptoms indicative of TB, were not housemates with TB patients, and had never been diagnosed with TB. The same comorbidities used to exclude TB patients were also used to exclude volunteers from the healthy control group.

Both study populations were between 18 and 56 years of age. The clinical characteristics of the study groups are summarized in Table 1.

**Table 1:** Characteristics of the study population

Characteristics	Healthy controls (*n* = 29)	TB patients (*n* = 33)	*p* value
Sex (male/female)	15/14	18/15	0.6
Age (years; median/range)	30 (20–50)	36 (18–56)	0.3
BCG vaccinate (%)	100	100	—
Chest X‐ray suggestive TB (%)	0	100	—
Chest X‐ray suggestive TB (%)	0	100	—
Previous TB (%)	0	0	—
Comorbidity (%)	0	0	—

### 2.2 Ethical approval

All procedures performed in the study were in accordance with the 1964 Helsinki Declaration as well as with the ethical standards of the *Instituto Nacional de Enfermedades Respiratorias Ismael Cosio Villegas*' Ethics Committee (No. B01‐06). Informed consent was obtained from all participants included in the study.

### 2.3 Culture filtrate antigen extract

Culture filtrate antigen extract (CFAE) was used as the mycobacterial antigen. It was obtained from the *Mtb* H37Rv strain according to standard procedures. In brief, bacteria were grown at 37°C in a Middlebrook 7H9 medium enriched with OADC (oleic albumin dextrose catalase; Difco Laboratories Inc., BD Diagnostic Systems, Detroit, MI, USA) until the mid‐logarithmic phase. Two rounds of filtration through 0.45 μm and 0.22 μm pore‐sized membranes (Merck Millipore Corp., Darmstadt, Germany) removed the bacillary material, after which the protein concentration was adjusted to 2 mg/ml and the samples were stored at −20°C until further use.^17^

### 2.4 Peripheral blood mononuclear cells

Peripheral blood mononuclear cells (PBMCs) were isolated from 20 ml of heparinized whole blood by Ficoll‐Hypaque (Sigma‐Aldrich, St Louis, MO, USA) density gradient centrifugation for 30 min at 500*g* and 16°C. After centrifugation, the interface cells were collected, washed three times in PBS (10 mm sodium phosphate, 150 mm sodium chloride, pH 7.2), and counted in a Neubauer chamber, determining cellular viability by a trypan blue‐dye exclusion method.

### 2.5 Multiparametric flow cytometry analysis

To measure the expression of different molecules on CD8^+^ cells, PBMCs were suspended in PBS containing 0.2% BSA and 0.1% sodium azide (PBS–BSA buffer). Cells were stained with the following monoclonal antibodies (mAbs): Phycoerythrin Cy5 (PECy5)‐CD8, Fluorescein isothiocyanate (FITC)‐TCRαβ, Phycoerythrin (PE)‐TCRγδ, PE‐NKG2D, FITC‐NKG2A, FITC‐CD69, PE‐CD25, PE‐CD56, FITC‐CD161, and PE‐CD16 (BD Biosciences Pharmingen, San Diego, CA), and then incubated for 20 min at 4°C. Data were collected using a FACSCalibur flow cytometer (BD Biosciences, San Jose, CA, USA) and analyzed by FlowJo software (Tree Star, San Carlos, CA, USA). In each case, 20,000 events were acquired. Fluorochrome‐labeled isotype‐matched control mAbs were used to evaluate background staining.

### 2.6 Cellular activation assay

PBMCs were seeded in 24 well, flat‐bottomed cell culture plates (Corning Costar Sigma‐Aldrich, St Louis, MO, USA) at a density of 1 × 10^6^ cells/well in an RPMI 1640 medium supplemented with 1 mm of sodium pyruvate, 2 mm of l‐glutamine, 50 μm of 2‐mercaptoethanol, 100 IU/ml of penicillin, 100 μg/ml of streptomycin, and 10% heat‐inactivated fetal calf serum (supplemented RPMI1640 medium) for 4 hr of stimulation by 25 ng of phorbol 12‐myristate 13‐acetate (PMA) and 1 μg of ionomycin (IO; Sigma‐Aldrich, St Louis, MO, USA) at 37°C in a humidified atmosphere containing 5% CO_2_.

### 2.7 Enrichment of monocytes and CD8^+^ T cells

PBMCs were suspended in PBS supplemented with 0.5% BSA and 2 mm of EDTA (1 × 10^7^ cells/0.1 ml) and incubated with microbeads coated with mAb CD14 (Miltenyi Biotech, Bergisch Gladbach, Germany) to obtain an enriched monocytes fraction by a positive selection system. The purity percentage of CD14^+^ cells was routinely 90%–95%, as measured by flow cytometry.

The negative fraction of CD14 was suspended in PBS supplemented with 0.5% BSA, and 2 mm of EDTA (1 × 10^7^ cells/0.1 ml) was incubated with microbeads coated with an mAb cocktail (CD4, CD15, CD16, CD19, CD34, CD36, CD56, CD123, and CD235a) by a negative magnetic selection kit according to manufacturer's instructions (Miltenyi Biotech, Bergisch Gladbach, Germany). The purity percentage of CD8^+^ T cells was habitually 93%–97%, as determined by flow cytometry.

### 2.8 Cell cytotoxicity assay

Cell‐mediated cytotoxicity was evaluated by flow cytometry as previously described.^18^, ^19^ In brief, purified, unstained CD14^+^ cells (target cells) were cultured in 96 well, flat‐bottomed cell culture plates with an ultra‐low attachment surface (Corning Costar Sigma‐Aldrich, St Louis, MO, USA) at a density of 1 × 10^5^/100 μl of supplemented RPMI‐1640 medium in the presence of different mycobacterial antigen (CFAE) concentrations (from 5 to 50 μg/ml) at 37°C in a 5% CO_2_‐humidified atmosphere.

Purified CD8^+^ T cells (effector cells) were labeled with a carboxyfluorescein diacetate succinimidyl ester (CFSE) dye at a concentration of 200 nm/1 × 10^6^ cells (prepared from a 5 mm stock solution dissolved in dimethyl sulfoxide).

The culture of the target cells and the mycobacterial antigen were incubated for 30 min (to intake antigens). Afterward, effector cells (CD8^+^CFSE^+^ cells) were added to the target cells in a 1:1 ratio (target:effector cells) at a final volume of 200 μl of RPMI1640‐supplemented culture medium.

The coculture was maintained in a humidified atmosphere containing 5% CO_2_ for 4 hr at 37°C. After the culture, the cells were harvested, washed in PBS, and incubated by a 0.25 μg 7‐amino actinomycin D (7‐AAD) solution for 20 min at 4°C in the dark. An extra condition with solitary target cells (without effector cells) was included to identify basal lysis. The percentage of dead cells was measured via 7‐AAD staining on the gated target cells (CFSE^–^ cells) by flow cytometry. Typically, 20,000 events were acquired for each condition. The analysis was performed with FlowJo (Tree Star) software.

To evaluate inhibition of antigen‐induced monocyte death, the coculture was incubated with both 20 μg/ml of CFAE and mAb 3 μg anti‐HLA‐A, B, C (Clone DX17), the culture was maintained in a humidified atmosphere containing 5% CO_2_ for 4 hr at 37°C. The optimal dose of mAb needed to block MHC‐A, B, C was obtained by staining of cells with fluorescent mAb to HLA‐A, B, C after incubation with different protein concentrations of HLA‐A, B, C mAb blockers. The absence of staining on monocytes indicated the optimal dose of HLA‐A, B, C mAb blocking. Isotype‐matched mAb to an unrelated molecule was used as experimental control.

### 2.9 Multiple cytokine assay

Plasmas were frozen at −20°C until use. IFN‐γ, TNF‐α, IL‐1β, IL‐8, IL‐4, and IL‐10 were measured by a Bio‐Plex multiplex cytokine assay kit according to manufacturer's instructions (Bio‐Rad Labs, Hercules, CA, USA). In brief, plasmas were centrifuged at 10,000*g* for 10 min at 4°C to remove precipitates and platelets. Bead‐coupled capture antibodies were added to each well of the plate, incubated for 10 min, and washed twice. Fifty microliters of standards and plasma were added to the wells, the plate is incubated, and shaken gently for 30 min at room temperature in the dark. After three washings, a biotinylated detection antibody was added to the wells, and the plates were incubated for 30 min. Finally, PE‐labeled streptavidin was added to the wells and incubated for 10 min. The plate was read on a Bio‐Plex Luminex 200 system. The different cytokine concentrations were calculated in picogram/milliliter using Bio‐Plex Manager version 6.0 software (Bio‐Rad Labs, Hercules, CA, USA). Standard curves for each cytokine were made by means of standards provided by the manufacturer.

### 2.10 Statistical analysis

Data were evaluated by GraphPad Prism 6 software (GraphPad Software, Inc., San Diego, CA, USA) using a Mann–Whitney *U* test to compare differences between two independent groups, and the values were shown as median ± minimum and maximum as the interquartile range (IQR). Differences between groups were considered statistically significant at *p* < 0.05.

## 3 RESULTS

### 3.1 Clinical characteristics of enrolled participants

Based on the clinical diagnosis, 62 participants were divided into two groups: 33 TB patients with a confirmed diagnosis of active pulmonary TB and 29 healthy controls. We did not perform the purified protein derivative test; however, in Mexico the BCG vaccine was made obligatory since 1973 as part of the national scheme for childhood vaccination. Thus, as is shown in Table 1, all of the patients received the BCG vaccine. The male‐to‐female ratio and the median age were similar between the groups; likewise, neither group presented comorbidities, such as lung cancer, HIV, or diabetes. Using the data from Table 1, we assumed that our final results were a direct effect of active TB infection and were not masked by either the presence of a second comorbidity or by differences in age or sex.

### 3.2 Increased percentage of peripheral blood CD8^+^αβ^+^ cells in TB patients

**Figure 1:**
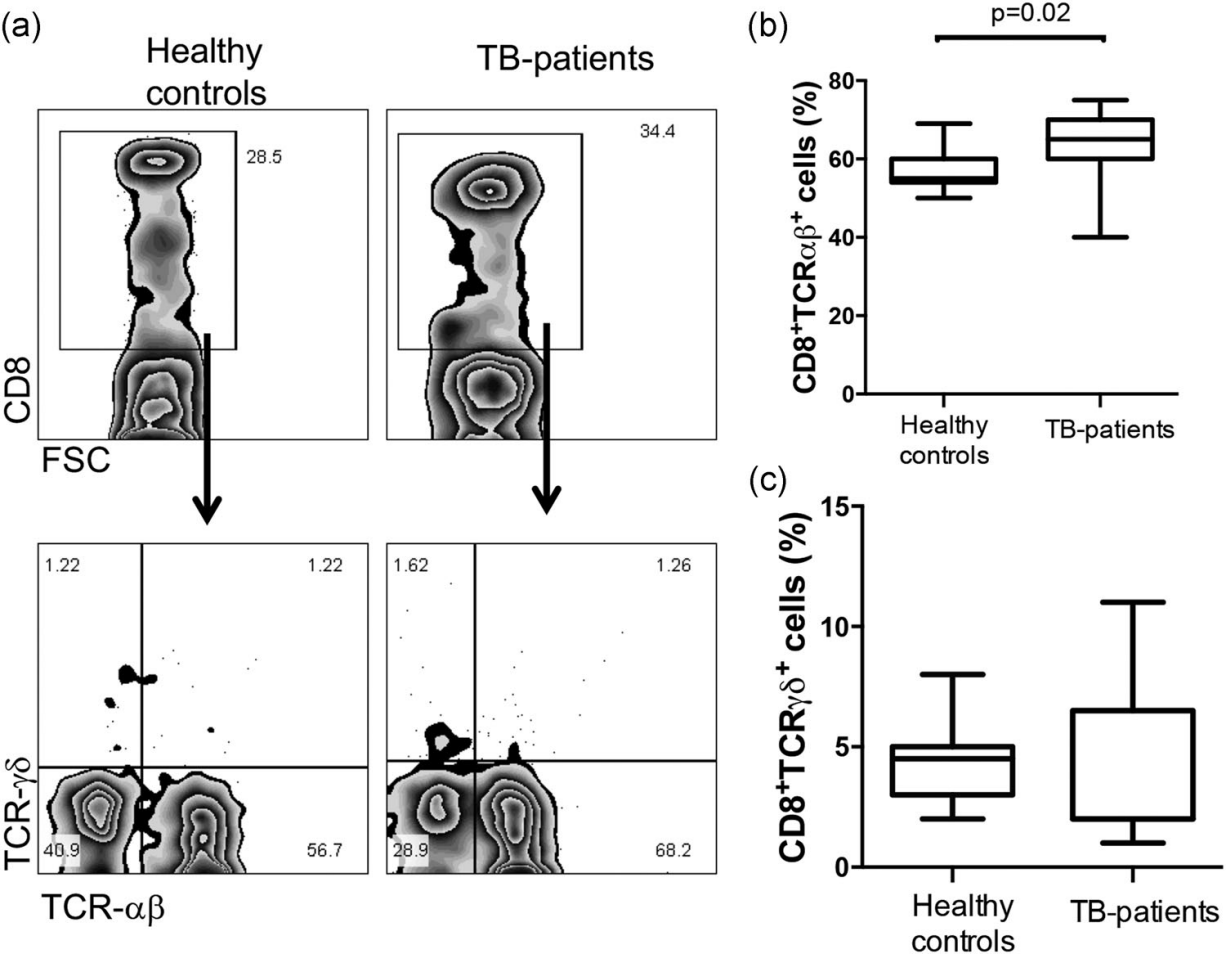
CD8^+^TCRαβ^+^ cells are increased in peripheral blood of TB patients. PBMCs from healthy controls and TB patients were stained and analyzed for cell surface markers. First, a gate was designed to include CD8^+^ cells; then, within gated CD8^+^ cells, the frequency of TCRαβ^+^ and TCRγδ^+^ cells was measured. Representative plots are shown (a). Data were collected on a FACSCalibur flow cytometer. Data are represented as box and whisker graphic plots, and horizontal bars represent the median value with a minimum and maximum range (b,c). Statistical analysis was performed using a Mann–Whitney *U* test, and the significant *p* value is indicated in the figure

It has been shown that TCRαβ^+^ and TCRγδ^+^ T cells facilitate a protective response in TB infection.^20^, ^21^ To analyze the frequency of TCRαβ^+^ versus TCRγδ^+^ on CD8^+^ cells, PBMCs from TB patients and healthy controls were used. We delimited CD8^+^ cells and, inside this gate, assessed TCR expression (Figure 1a). In Figure 1b, it is shown that TB patients have an increased percentage of peripheral blood CD8^+^TCRαβ^+^ cells (median 55% IQR 50–69 vs. 65% IQR 40–75, *p* = 0.02). However, the frequency of peripheral CD8^+^TCRγδ^+^ blood cells in TB patients was no different from that of healthy controls (median 4% IQR 2–8 vs. 2% IQR 1–11) (Figure 1c).

### 3.3 TB patients have decreased NKG2D^+^CD8^+^ cells but increased CD56^+^CD8^+^ cells frequency

**Figure 2:**
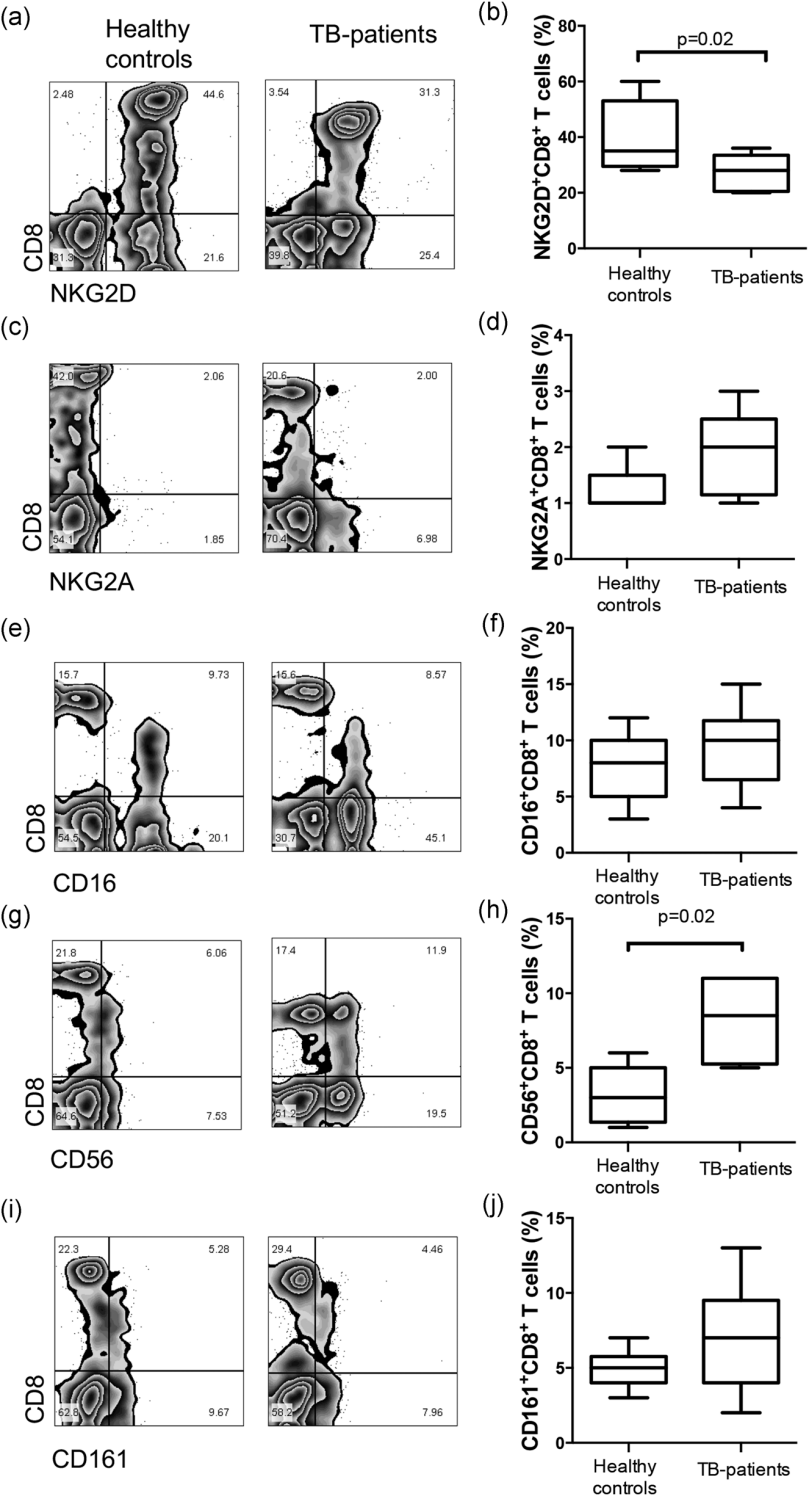
Frequency of NKG2D^+^CD8^+^ cells is decreased in peripheral blood of TB patients. PBMCs from healthy controls and TB patients were stained and analyzed for cell surface markers, and CD8 versus NKG2D, NKG2A, CD16, CD56, and CD161 were measured. Representative plots show coexpression of CD8 with NKG2D, KNG2A, CD16, CD56, and CD161 (a,c,e,g,i, respectively). Data were collected on a FACSCalibur flow cytometer. Data are represented as box and whisker graphic plots, and horizontal bars represent the median value with a minimum and maximum range (b,d,f,h,j). Statistical analysis was performed using a Mann–Whitney *U* test, and the significant *p* value is indicated in the figure

NKG2D is an activating receptor expressed on the surface of NK cells and CD8^+^ T cells. The NKG2A inhibitor receptor is expressed on T cells only under specific conditions and after a long culture time.^5^, ^22^ In PBMCs from TB patients and healthy controls, we identified the frequency of CD8^+^ cells that coexpress NKG2D and NKG2A (Figure 2a,c, respectively). Our data show that TB patients had a decreased percentage of NKG2D^+^CD8^+^ cells (median 35% IQR 28–60 vs. 28% IQR 20–38, *p* = 0.02; Figure 2b), but the percentage of CD8^+^ cells that coexpressed the NKG2A inhibitor receptor were similar in both study populations (median 2% IQR 1–3 vs. 1% IQR 1–2; Figure 2d).

To identify whether CD8^+^ cells coexpressed other markers, such as CD16, CD56, and CD161, an analysis by flow cytometry was performed (Figure 2e,g,i, respectively). In Figure 2h, an increased proportion of CD56^+^CD8^+^ subpopulation cells in peripheral blood from TB patients can be seen in comparison with those from the healthy control group (median 3% IQR 1–6 vs. 9% IQR 5–11, *p* = 0.02). By contrast, the frequencies of CD16^+^CD8^+^ and CD161^+^CD8^+^ cells were similar in both study populations (median 8% IQR 3–12 vs. 10% IQR 4–15; and median 5% IQR 3–7 vs. 7% IQR 2–13, respectively; Figure 2f,j). It is important to note that CD8^+^ cells that coexpress CD16 markers correspond to a subpopulation of cytotoxic T cells that display low CD8 expression, frequently called CD8^low^; however, CD56 expression on CD8^+^ cells from TB patients is not limited to CD8^low^, as there is also a CD8^high^ fraction which expresses CD56 (Figure 2f,h).

### 3.4 CD8^+^ cells from TB patients and healthy controls have the same ability to activate with a polyclonal stimulus

**Figure 3:**
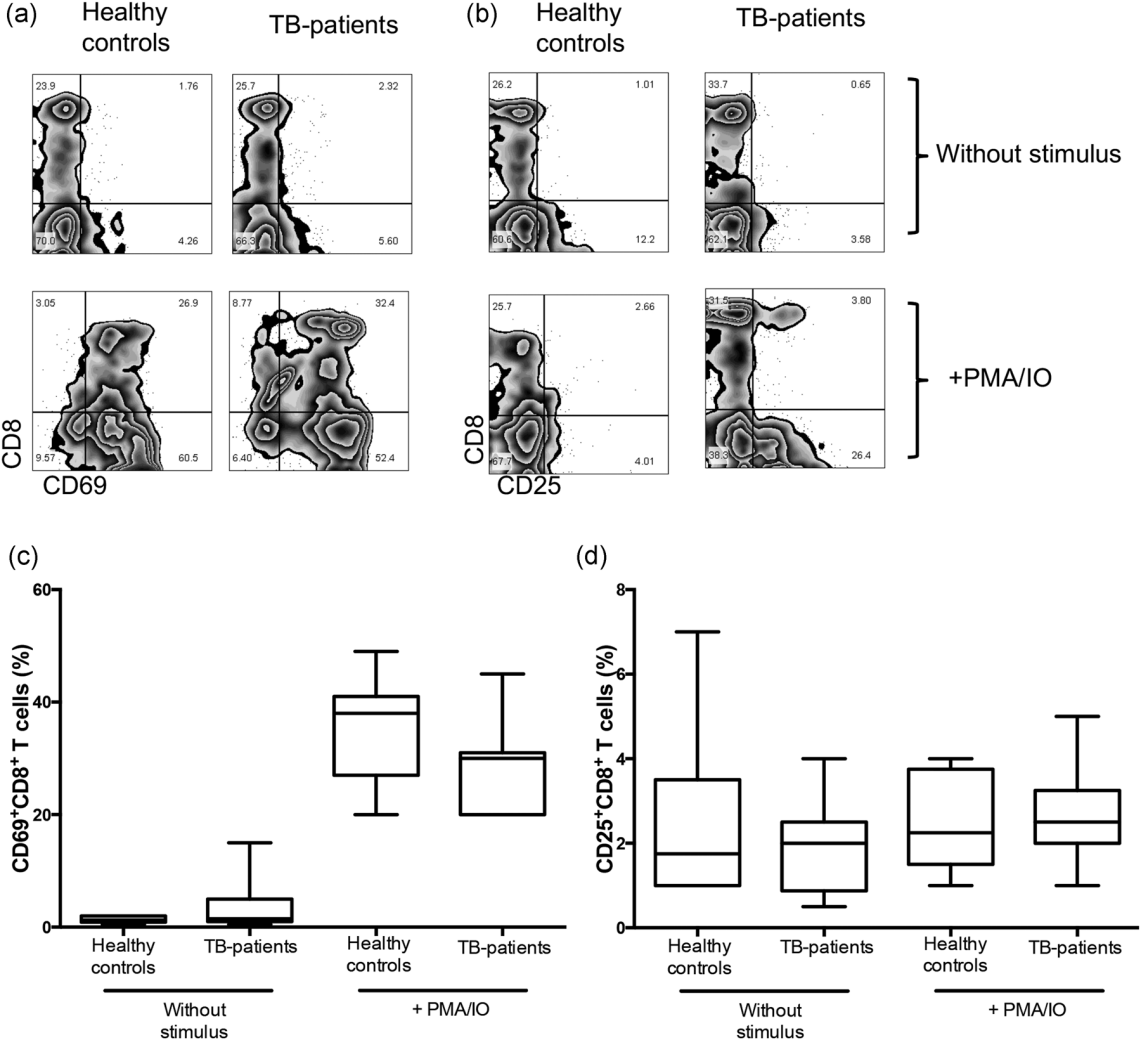
CD8^+^ cells from TB patients are efficient to activate in response to mitogenic stimulus. PBMCs from healthy controls and TB patients were incubated with PMA/IO at a final concentration of 25 ng/1 μg per 1 × 10^6^ cells for 4 hr at 37°C. After the culture, cells were stained and analyzed for cell surface markers, and CD8 versus CD69 and CD25 were measured. Representative plots show the coexpression of CD8 with (a) CD69 and (b) CD25. Data were collected on a FACSCalibur flow cytometer. Data are represented as box and whisker graphic plots, and horizontal bars represent the median value with a minimum and maximum range (c,d). Statistical analysis was performed using a Mann–Whitney *U* test, and the significant *p* value is indicated in the figure

To obtain insights into the activation ability of CD8^+^ cells in TB patients, we performed a cell culture in the presence of a polyclonal stimulus (PMA/IO, 4 hr), and CD69 and CD25 expression on CD8^+^ T cells were evaluated (Figure 3a,b, respectively).^23^, ^24^

Before the culture, the frequency of CD8^+^ cells that coexpressed CD69 was low in both study groups (median healthy controls 1% IQR 0.5–2 and 2% IQR 0.5–15 in TB patients; Figure 3c). As was expected, after 4 hr in the culture with PMA/IO, the percentage of CD69^+^CD8^+^ cells increased in both healthy donors and TB patients (median 38% IQR 20–49 vs. 30% IQR 20–45; Figure 3c). Regarding the coexpression of CD25 on CD8, our data show that the frequency of CD8^+^CD25^+^ cells was practically absent without a stimulus (median healthy controls 2% IQR 1–7 vs. 2% IQR 0.5–4 in TB patients; Figure 3d); in accordance with previous reports, the percentage of CD8^+^CD25^+^ cells was still low in the first hours following polyclonal stimulus (median healthy controls 2% IQR 1–4 vs. 2% IQR 1–5 in TB patients; Figure 3d).

### 3.5 Multiparametric analysis of lysed target cells

**Figure 4:**
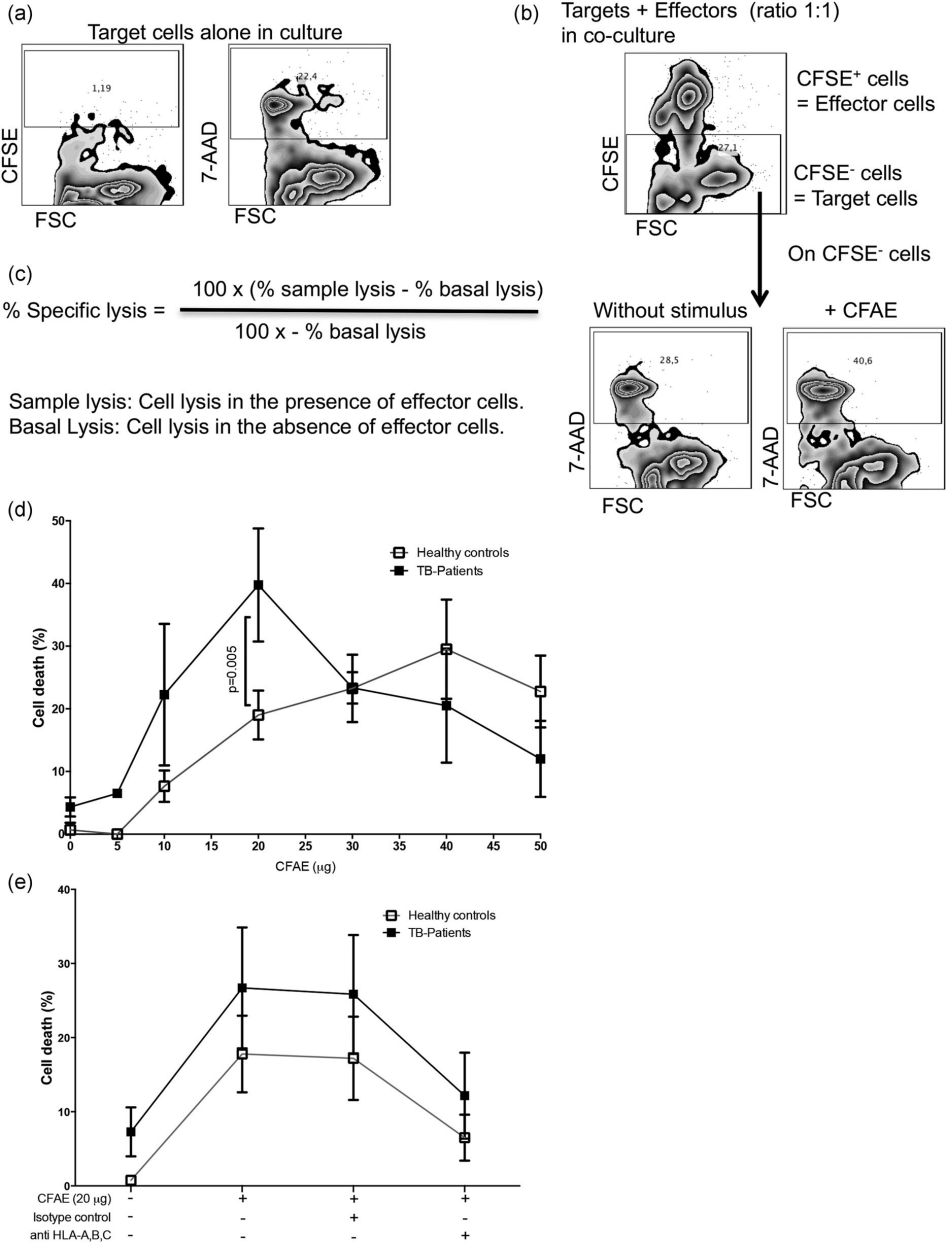
Cytotoxicity assay by flow cytometry demonstrated that CD8^+^ T cells from TB patients are highly cytotoxic in response to CFAE stimulus. Monocytes and CD8^+^ T cells were isolated from mononuclear peripheral blood cells by magnetic bead‐labeled antibodies, incubated with CFAE, and analyzed by flow cytometry. The condition of monocytes being alone in the culture was necessary to delimit the negative gate to CFSE, then inside of CFSE‐ gate, 7‐AAD staining percentage was obtained as measure of manocytes basal lysis (antigen‐ and effector cells‐ independent) (a). After staining CD8^+^ cells with CFSE (200 nm), they were put in a coculture with monocytes and delimited CFSE^–^ cells (monocytes or target cells); each different condition evaluated the percentage of 7‐AAD^+^ to calculate the percentage of specific lysis (b and c, respectively). Monocytes and CD8^+^ T cells were isolated from mononuclear peripheral blood cells by magnetic bead‐labeled antibodies, incubated with CFAE, and analyzed through flow cytometry. A coculture was conducted for 4 hr at 37°C using a dose–response curve with a CFAE stimulus from 0 to 50 μg (d). Other experiments were performed using 20 μg of CFAE plus mAb anti‐HLA‐A, B, C (3 μg) (e). After the culture, cells were stained with 7‐AAD. The analysis was completed as described for the cytotoxicity assay by flow cytometry. Data were collected on a FACSCalibur flow cytometer. Data represent the median value of five independent experiments, and the symbols represent the median and standard deviation. Statistical analysis was performed using a Mann–Whitney *U* test, and the significant *p* value is indicated in the figure

We used a flow cytometry method that combined two dyes: CFSE to label effector cells, and 7‐AAD to characterize cell death. To identify the percentage of antigen‐specific lysis, we used a previously reported method. In Figure 4, we included the analysis strategy by flow cytometry to clarify how the percentage of specific lysis was obtained.^18^ One condition of the cellular culture only had monocytes (target cells) and was used to obtain the negative control for CFSE staining and the basal lysis, this mean that the basal lysis is independent of antigen or the presence of effector cells (Figure 4a). Another condition for the cellular culture was that it was obtained with a coculture of target cells plus CFSE‐stained effector cells in a 1:1 ratio. The CFSE^–^ gate was obtained to identify target cells (Figure 4b, upper panel). Inside the CFSE^–^ region, the percentage of 7‐AAD^+^ cells was measured. These cells were monocytes that died as a consequence of the presence of effector cells in the absence or presence of the antigen (Figure 4b, lower, left, and right panels, respectively). Finally, the percentage of specific lysis was obtained using the calculation shown in Figure 4c.

### 3.6 Increased cytotoxic ability by CD8^+^ cells from TB patients

Our data show that CD8^+^ T cells from TB patients had a higher level of cytotoxic activity using 20 μg of CFAE (median healthy control, 19% vs. TB patients, 40%, *p = *0.005); however, the healthy group required 40 μg of CFAE to produce the maximum level of cytotoxic function (median healthy control, 28% vs. TB patients, 21.5%). In fact, 10 μg of CFAE CD8^+^ from TB patients induced 2.5 times more cytotoxic activity compared with CD8^+^ T cells from healthy donors, but we were not able to identify any statistical difference (median 8% vs. 20%, *p = *0.07) (Figure 4d).

Considering that CD8^+^ T cells from TB patients coexpress NK molecules, such as CD56, and induce cytotoxic activity with a lower antigen concentration, we evaluated whether this cytotoxic activity was induced by the classical HLA‐A, B, C pathway. The percentage of cell death induced without the antigen was similar to that previously obtained in Figure 4d (median 1% vs. 8%). By adding 20 μg of CFAE, the percentage of dead cells increased, as was expected (median 17% vs. 30%), and the use of isotype‐matched mAb did not affect the cytotoxic activity of CD8^+^ T cells (median 16% vs. 26%; Figure 4e). However, in the cellular coculture condition to which anti HLA‐A, B, C was added, it was found that the percentage of dead cells decreased in both the healthy controls and the TB patients (median 7% vs. 11%; Figure 4e). These data suggest that CD8^+^ T cells from TB patients have high cytotoxicity; they can induce cell death with lower antigen doses than those from healthy controls, and this activity is mediated by the classical class I pathway HLA.

### 3.7 TNF‐α, IL‐8, and IL‐4 concentrations are increased in plasma from TB patients

**Figure 5:**
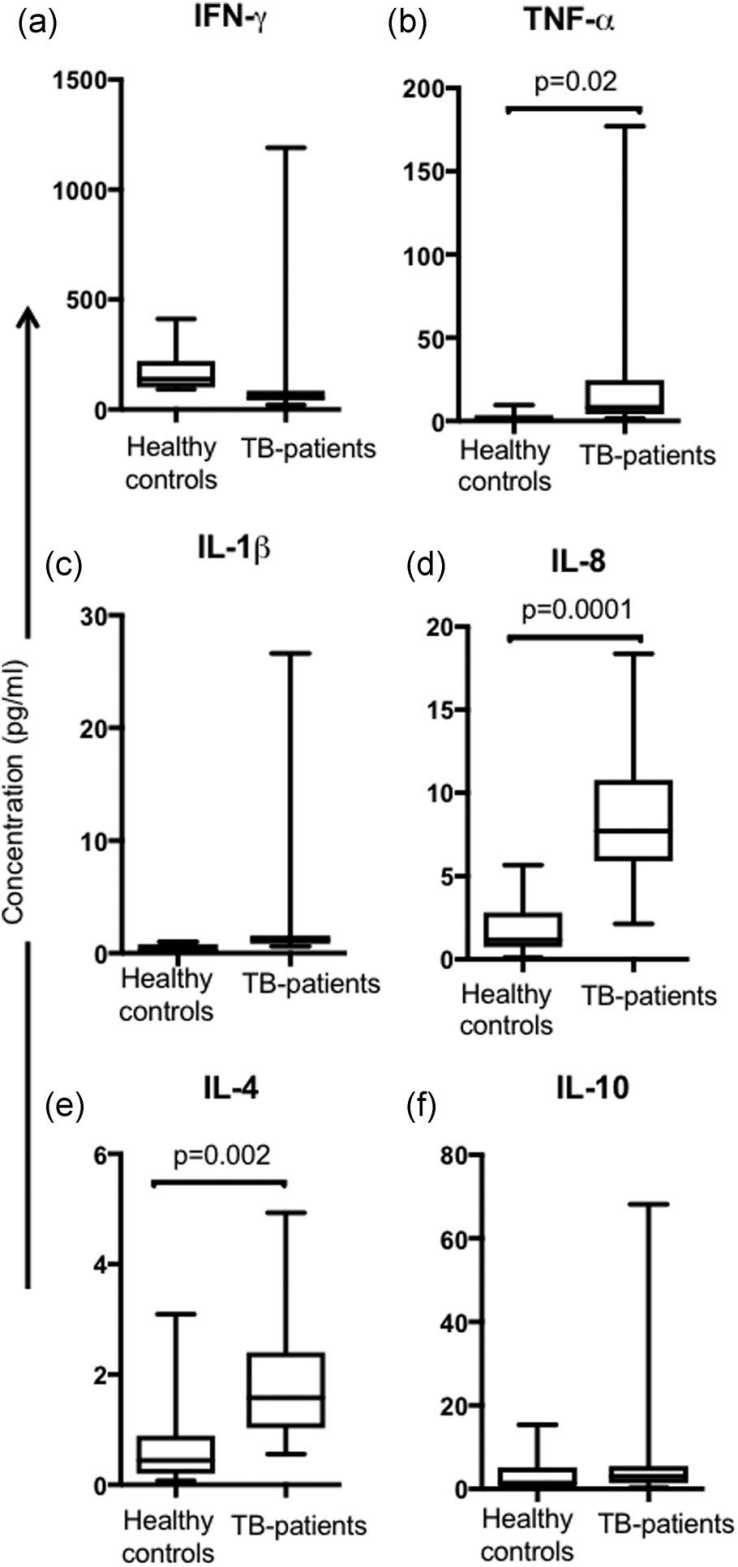
TB patients have a high level of TNF‐α, IL‐8, and IL‐4. Plasma samples from 10 healthy controls and 15 TB patients were obtained. Using a Bio‐Plex magnetic bead‐based kit, IFN‐γ, TNF‐α, IL‐1β, IL‐8, IL‐4, and IL‐10 levels were measured (from a to f). Data are represented as box and whisker graphic plots, and horizontal bars represent the median value with a minimum and maximum range. Statistical analysis was performed using a Mann–Whitney *U* test, and the significant *p* value is indicated in the figure

The role of cytokines in the immune response to *Mtb* infection is well established.^25^ We evaluated the cytokine profile in the serum from TB patients to clarify the microenvironment in which the cells are maintained *in vivo*. We measured proinflammatory (IFN‐γ, TNF, IL‐1β, IL‐8) and anti‐inflammatory (IL‐4 and IL‐10) cytokines. TB patients, compared with healthy controls, have increased serum levels of TNF (median healthy controls 1 IQR 0.2–10 vs. TB patients 8 IQR 2–177 pg/ml, *p* = 0.02), IL‐8 (median healthy controls 1 IQR 0.1–6 vs. TB patients 8 IQR 2–18 pg/ml, *p = *0.0001), and IL‐4 (median healthy controls 0.4 IQR 0.07–3 vs. TB patients 2 IQR 0.5–pug/ml, *p* = 0.002; Figure 5b,d,e, respectively). However, we were not able to identify any differences in the concentrations of IFN‐γ (median healthy controls, 140 vs. TB patients, 411 pg/ml), IL‐1β (median healthy controls, 0.2 vs. TB patients, 1 pg/ml), and IL‐10 (median healthy controls, 1 vs. TB patients, 3 pg/ml; Figure 5a,c,f, respectively). Together, these data show that TB patients exhibited an unregulated profile of cytokines in serum, whereas TNF‐α, IL‐8, and IL‐4 all increased.

## 4 DISCUSSION

At present, TB prevalence and mortality are still high, and epidemiology reports show that there is an urgent need to develop more efficient vaccines, treatments, and early markers of the disease; however, to do so, a better understanding of the immune mechanisms that determine the outcome of *Mtb* infection is required. Recent evidence suggests that CD8^+^ T cells play a critical role in controlling this infection.

To evaluate CD8^+^ cells from TB patients, the expression patterns of molecules, which are important in regulating cytotoxic activity, and their cytotoxic ability were measured. We demonstrated that CD8^+^ cells from TB patients have a high frequency of CD8^+^αβ^+^cells. CD56 and NKG2D expression were altered, and they were highly cytotoxic, principally by restricted HLA‐A, B, C.

Our data show that TB patients have a high frequency of CD8^+^ cells; even if we do not evaluate TCR repertoires, our data are still in agreement with previous reports that described the clonal expansion of CD8^+^ TCRαβ^+^ T cells in children and in the macaque model.^9^, ^15^ Because CD8^+^ TCRγδ^+^ cells were not increased, we assume that the cytotoxic activity of CD8^+^ TCRαβ^+^ cells against *Mtb* could be the most important mechanism in infection control.

Before evaluating the function of CD8^+^ cells, we measured the expression of molecules that were shared between the main cytotoxic cells (NK and CD8^+^ T cells). Our data showed that the frequency of NKG2D^+^CD8^+^ T cells decreased, but that of the CD56^+^CD8^+^ T cells increased. The expression of other molecules, such as NKG2A, CD16, and CD161, were not modified (Figure 2). *Mtb* infection was not found to decrease the NKG2D expression of CD8^+^ T cells. However, in cancer models, the decreased expression of NKG2D both on CD8^+^ T and on NK cellular surfaces was demonstrated, and it was determined to be a mechanism that compromises cell function and promotes tumor immune escape.^26^, ^27^ Regarding human CD56^+^CD8^+^ T cells, it has been demonstrated that this subpopulation is less vigorous in terms of proliferation, after which it is bound to classical class I HLA. This subpopulation displays enhanced natural cytotoxicity activity and cytokine secretion, and it was proposed that the frequency of CD56^+^CD8^+^ T cells could be affected by cytomegalovirus infection and aging.^28^, ^29^ With this phenotypic characterization, we provided information about how CD8^+^ cells from TB patients could be NKG2D downregulated as an evasion system, probably by favoring *Mtb* infection. However, the frequency of CD56^+^CD8^+^ T cells increased, indicating that NK markers are regulated opposite to CD8^+^ cells in TB patients.

To test the hypothesis that cytolytic function would be affected in CD8^+^ T cells, we adopted a cellular coculture system and used a flow cytometry technique to evaluate their functionality. We showed that CD8^+^ T cells from TB patients are highly cytotoxic; these cells start their cytolytic function from a low dose of CFAE (Figure 4d), whereas CD8^+^ T cells from healthy donors require double the CFAE concentration to induce their maximum cytolytic ability. These results could be considered in the development of more efficient treatments. It was recently reported that the activation threshold and TCR affinity of CD8^+^ T memory cells should be the goal of vaccination against TB.^30^ These results therefore also support the relevance of CD8^+^ T cells in the vaccine field.

Interestingly, nearly all cytotoxic activity was blocked using anti‐HLA‐A, B, C. It is possible that under *Mtb* infection, CD8^+^ T cells restrict their function through the granule exocytosis pathway. As is shown in Figure 4e, cells from TB patients have a higher percentage of cell death in the absence of a mycobacterial antigen. A previous report suggested that monocytes from TB patients are more susceptible to death,^19^ and this probably explains the high activation threshold without CFAE.

CD8^+^ T cells called “polycytotoxic T cells” were recently defined and are present in bronchoalveolar lavage cells in TB patients. They are lipoarabinomannan (LAM)‐responsive T cells that contribute to disease outcome.^31^ By contrast, it has been demonstrated that TB patients have LAM in their peripheral blood.^32^ We suggest that the high cytotoxic ability shown by CD8^+^ T cells in TB patients could be a consequence of the frequent interaction of LAM–CD8^+^ T cells in peripheral blood. Using an *in vitro* model, it has been demonstrated that LAM has a direct effect on cells, affecting cellular differentiation and functionality.^33^ It has recently been suggested that the generation of TB vaccines carrying CD8^+^ T cells stimulates antigens that have the potential to prevent the progression of latent TB infection to TB diseases.^34^ Thus, our results provide new knowledge regarding the status of CD8^+^ cells in peripheral blood under *Mtb* infection.

In agreement with previous reports, TNF‐α and IL‐8 levels were increased in TB patients. In fact, these mycobacterial‐specific cytokines have been proposed for use in immunodiagnostics.^35^, ^36^ Interestingly, our data lend support to the theory that TB patients have a Th1/Th2 imbalance that is characterized by high IL‐4 but low IFN‐γ levels, a phenomenon that is a significant factor in the pathogenesis and development of TB.^37^, ^38^ It is possible that this Th1/Th2 balance shift plays a role in the high cytotoxic background of CD8^+^ T cells.

This study was limited in that it was not longitudinal. Thus, it was not possible to determine whether a high frequency of CD8^+^αβ^+^ cells remained in peripheral blood after TB treatment or whether their cytotoxic ability was modified. However, previous reports can help us to obtain a possible answer to these questions. In 2013, it was shown that *Mtb* protein‐specific CD8^+^ T cells decline with anti‐TB treatment, suggesting that this decrease could be a surrogate marker in response to therapy. Previously, Day et al showed that *Mtb* protein‐specific CD8^+^ T cells from TB patients have a proapoptotic phenotype and impaired proliferative capacity, and this could explain the decline in their frequency after treatment.^39^, ^40^

In conclusion, our study highlights the cytotoxic response against *Mtb* infection. We demonstrated that CD8^+^ T cells from TB patients are principally TCRαβ^+^, and that even when they have a downregulated NKG2D expression, CD56^+^ is upregulated. Finally, CD8^+^ T cells from TB patients are highly cytotoxic. There is thus a need to expand research on the presence and function of CD8^+^ T cells in peripheral blood from TB patients to better understand the immune response against *Mtb*, identify the activation pathway needed to eliminate *Mtb*, optimize strategies for designing new epitopes targeted by mycobacterium‐specific CD8^+^ T cells, obtain new TB vaccines with better protective immunity than current vaccines, or identify the markers of disease progression.

## FUNDING INFORMATION

The present study was supported by Grant 82434 (to RL) from the Consejo Nacional de Ciencia y Tecnología, Mexico.

## DISCLOSURE

The authors declare that they have no conflict of interests related to this work.
